# Novel proteolytic activation of Ebolavirus glycoprotein GP by TMPRSS2 and cathepsin L at an uncharted position can compensate for furin cleavage

**DOI:** 10.1016/j.virusres.2024.199430

**Published:** 2024-07-08

**Authors:** Dorothea Bestle, Linda Bittel, Anke-Dorothee Werner, Lennart Kämper, Olga Dolnik, Verena Krähling, Torsten Steinmetzer, Eva Böttcher-Friebertshäuser

**Affiliations:** a Institute of Virology, Philipps-University, Marburg, Germany; b German Center for Infection Research (DZIF), Partner Site Gießen-Marburg-Langen, Marburg, Germany; c Institute of Pharmaceutical Chemistry, Philipps-University Marburg, Germany

**Keywords:** Ebola virus, GP cleavage, Furin, Endosomal cathepsins, TMPRSS2

## Abstract

A multistep priming process involving furin and endosomal cathepsin B and L (CatB/L) has been described for the Orthoebolavirus zairense (EBOV) glycoprotein GP. Inhibition or knockdown of either furin or endosomal cathepsins, however, did not prevent virus multiplication in cell cultures. Moreover, an EBOV mutant lacking the furin cleavage motif (RRTRR→AGTAA) was able to replicate and cause fatal disease in nonhuman primates, indicating that furin cleavage may be dispensable for virus infectivity. Here, by using protease inhibitors and EBOV GP-carrying recombinant vesicular stomatitis virus (VSV) and transcription and replication-competent virus-like particles (trVLPs) we found that processing of EBOV GP is mediated by different proteases in different cell lines depending on the protease repertoire available. Endosomal cathepsins were essential for EBOV GP entry in Huh-7 but not in Vero cells, in which trypsin-like proteases and stably expressed trypsin-like transmembrane serine protease 2 (TMPRSS2) supported wild-type EBOV GP and EBOV GP_AGTAA mutant entry. Furthermore, we show that the EBOV GP_AGTAA mutant is cleaved into fusion-competent GP_2_ by TMPRSS2 and by CatL at a so far unknown site. Fluorescence microscopy co-localization studies indicate that EBOV GP cleavage by TMPRSS2 may occur in the TGN prior to virus release or in the late endosome at the stage of virus entry into a new cell.

Our data show that EBOV GP must be proteolytically activated to support virus entry but has even greater flexibility in terms of proteases and the precise cleavage site than previously assumed.

## Highlights

• The necessity of EBOV GP cleavage at the furin cleavage site was and is a subject of debate.
• The furin cleavage site mutant EBOV GP_AGTAA, which was described as non-cleavable, is shown to be cleaved by TMPRSS2 and cathepsin L.
• IF suggests that TMPRSS2 may cleave EBOV GP upon entry in the late endosome or at later stages in the TGN.
• Proteolytic activation of EBOV GP offers even greater flexibility than previously assumed.

## 1 Introduction

The genus *Orthoebolavirus* belongs to the order of *Mononegavirales* and to the family of *Filoviridae* (Biedenkopf et al., 2023). First discovered in 1976 in Central Africa, until now six species with their representative viruses (*Ebola virus* (EBOV), *Sudan virus* (SUDV), *Taї Forest virus* (TAFV), *Reston virus* (RESTV), *Bundigugyo virus* (BDBV) and *Bombali virus* (BOMV)) have been identified. Ebola viruses cause the Ebola virus disease, that can lead to severe illness with hemorrhagic fever and an often fatal outcome in humans and non-human primates (Feldmann and Geisbert, 2011). EBOV has been associated with reccurring outbreaks in Central and West Africa with case fatality rates ranging from 25 to 90 %, with the largest outbreak occurring from 2013 to 2016 in West Africa (Sierra Leone, Guinea and Liberia) that was caused by the EBOV Makona strain and led to a total of 28.652 cases with 11.325 deaths (CDC 2019).

**Fig. 1:**
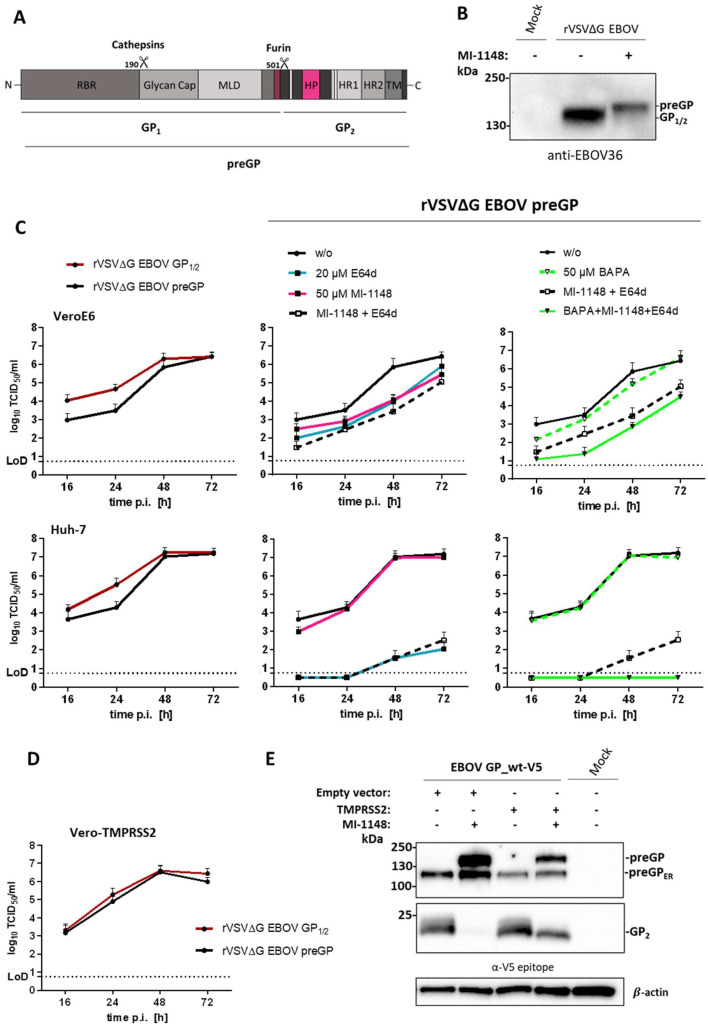
Replication of rVSV∆G EBOV GP_wt expressing uncleaved (preGP) glycoprotein in Vero and Huh-7 cells. A) Schematic illustration of EBOV GP surface protein domain structure. The GP is a type I transmembrane protein consisting of 676 aa. The receptor binding region (RBR) is located at the N-terminal end followed by the glycan cap and the mucin-like domain (MLD). The domains responsible for viral fusion, including the hydrophobic patch (HP) and the two heptad repeat domains (HR1 and HR2), are located at the C-terminal end of the protein prior to the transmembrane domain (TM). The EBOV GP is synthesized as an inactive precursor protein (preGP) that must be proteolytically activated ("primed") in order to gain its fusion capacity. Priming of preGP is a multistep process starting with an initial cleavage of preGP at R501 into the GP_1_ and GP_2_ subunits (GP_1/2_). GP_1_ is then further trimmed by endosomal cysteine proteases cathepsin B and L, leading to a loss of MLD and glycan cap domain and exposure of the RBR. B) GP expression on virus particles of furin-cleaved VSV∆G EBOV GP_1/2_ and uncleaved VSV∆G EBOV preGP. Both rVSV∆G EBOV GP_1/2_ and preGP virus stocks were propagated in Huh-7 cells. For the generation of VSV∆G bearing uncleaved preGP on the surface, cells were incubated with 30 µM of MI-1148 for 24 h. Virus supernatants were then subjected to an 8 % SDS-PAGE and analyzed with an EBOV-specific serum by western blot analysis. C) Multicycle replication of rVSV∆G EBOV GP_1/2_ or preGP in untreated VeroE6 and Huh-7 cells (left panels) or under single and combined inhibitor treatment with E64d, MI-1148 and BAPA (middle and right panels). Cells were treated with 50 µM MI-1148, 20 µM E64d and/or 50 µM BAPA for 1 h prior to rVSV∆G EBOV preGP infection or were left untreated. Afterward, the cells were inoculated with rVSV∆G EBOV preGP at a MOI of 0.005 for 1 h. Protease inhibitor treatment was continued during 72 h incubation period post infection (p.i.). Virus supernatants were collected at 16, 24, 48 and 72 h p.i. Viral titers were determined by TCID_50_ endpoint dilution assay. Data shown are means (+SD) of three to five independent experiments (*n* = 3–5). LoD: limit of detection. D) Comparative growth kinetics of rVSV∆G EBOV GP_1/2_ or preGP in Vero-TMPRSS2 cells. Infections were performed without prior inhibitor treatment and viral titers were analyzed as described above. Data shown are means (+SD) of five independent experiments (*n* = 5). E) Proteolytic cleavage of EBOV GP_wt with a C-terminal V5-tag (EBOV GP_wt-V5) by human TMPRSS2 in the absence of endogenous furin. HeLa cells were co-transfected with EBOV GP_wt-V5 and human TMPRSS2 plasmid for 24 h with or without MI-1148 (50 µM) treatment, empty vector transfection served as control. The cells were then harvested, lysed and the samples subjected to a 10% SDS-PAGE and immunoblotting. EBOV GP cleavage forms were detected with an antibody directed against the C-terminal V5 epitope of EBOV GP. ß-Actin staining was used as a loading control. preGP: uncleaved precursor; preGP_ER_: uncleaved precursor in endoplasmic reticulum (ER). The western blot shown is a representative immunoblot of three independent experiments (*n* = 3).

EBOV is an enveloped virus with the sole viral surface glycoprotein GP embedded as a trimer into the lipid membrane of the filamentous virion (Feldmann et al., 1993). Fusion of the viral lipid envelope and the host cell membrane mediated by viral fusion proteins is a prerequisite for enveloped viruses in order to release the viral genome into the host cell and thus essential for replication and infectivity (Klenk et al., 1975). The EBOV GP is synthesized as a 676 amino acid (aa) fusion-incompetent precursor protein (preGP) (Fig. 1A) that is proteolytically cleaved by furin along the secretory transport pathway within the trans-Golgi network (TGN) at a multibasic RRTRR↓ motif (aa 497 to 501) (Volchkov et al., 1998; Wool-Lewis and Bates, 1999). This initial priming step leads to the separation of the GP1 and GP2 subunits, which remain covalently linked by a disulfide bond between the cysteines (C) 53 and C609. Furin cleavage of GP is remarkably efficient, since unprocessed GP is not present on Ebola virions in any significant amount (Volchkov and Klenk, 2018). The GP1 subunit harbors the receptor binding region (RBR) that is initially masked by the N-glycosylated glycan cap region (aa 228–313) and the heavily N- and O-glycosylated mucin-like domain (MLD) (aa 314–464). The GP2 subunit consists of the classical class I fusion protein domain structures with the N- and C-terminal heptad repeat regions (HR1 and HR2), as well as the transmembrane domain (TM). Unlike most class I fusion proteins, the GP2 consists of an internal fusion loop that is formed by a disulfide bridge between C511 and C556 bearing the hydrophobic patch (HP) at the tip, instead of a fusion peptide (FP) at the N-terminal end (Jeffers et al., 2002; Gregory et al., 2011; Lee and Saphire, 2009). The cleaved GP1/2 is transported to the host cell membrane and subsequently incorporated into newly budding filamentous particles. After the internalization of EBOV particles by macropinocytosis into a new host cell the GP1 subunit is consecutively trimmed from a 50 kDa fragment to a 19 kDa product by endosomal cathepsin B or L (CatB and CatL), with final processing at position 190 (Dube et al., 2009), thereby stepwise removing the bulky MLD and glycan cap (Chandran et al., 2005; Schornberg et al., 2006; Kaletsky et al., 2007; Brecher et al., 2012). The proteolytic processing of GP1 by endosomal cathepsins leads to the exposure of the RBR, which then binds to the Niemann-Pick disease, type C1 (NPC1) cholesterol transporter that serves EBOV as receptor in the late endosome or lysosome (Wang et al., 2016; Carette et al., 2011; Côté et al., 2011). The endosomal receptor interaction is essential for the fusion of the viral and endosomal host membrane, however, receptor binding alone is not sufficient for fusion pore formation. Recent studies show that calcium ions (Ca2+) and further CatB activity enhance EBOV fusion in the late endosome, whereby the latter in particular is not yet understood (Carette et al., 2011; Côté et al., 2011; Odongo et al., 2023; Das et al., 2020).

Even though the proteolytic processing of EBOV GP into GP_1_ and GP_2_ by furin and GP_1_ trimming by endosomal CatB and CatL was shown in several publications (Volchkov et al., 1998; Wool-Lewis and Bates, 1999; Chandran et al., 2005; Schornberg et al., 2006; Kaletsky et al., 2007; Brecher et al., 2012), the necessity of host cell protease activation of EBOV GP has been a subject of discussion for a long time. EBOV GP was shown to be proteolytically cleaved in LoVo cells deficient for expression of functional furin (Wool-Lewis and Bates, 1999). Studies with recombinant EBOV possessing GP with a mutated furin cleavage site, due to substitution of the multibasic RRTRR motif to the non-basic AGTAA sequence, showed only an initial reduction of virus replication in VeroE6 cells. Furthermore, the infectivity and virulence of EBOV bearing the "non-cleavable" GP mutant was not impaired regarding the ability to infect non-human primates (Neumann et al., 2002; Neumann et al., 2007). Additionally, neither *in vitro* inhibition of cathepsins nor *in vivo* knockout of CatB or CatL in mice had an adverse effect on EBOV replication and infectivity (Marzi et al., 2012). Both studies suggested that proteolytic activation of EBOV GP by either furin or endosomal cathepsins is dispensable for virus replication. This raised the general question whether other proteases are involved in GP cleavage or whether EBOV GP cleavage is even dispensable for its fusion activity. However, processing of other viral class I fusion proteins such as the influenza A virus hemagglutinin or the SARS-CoV-2 spike protein has been demonstrated to be essential for viral replication and infectivity in several human and murine *in vitro* models (Klenk et al., 1977; Limburg et al., 2019; Bestle et al., 2021; Bestle et al., 2024; Li et al., 2021), leaving the question whether EBOV GP is a unique member of the class I fusion proteins that is able to replicate independently of host cell protease activation.

In this work, we have investigated the proteolytic activation of the GP of EBOV (C7 Makona), with special focus on the AGTAA furin cleavage site mutant, on protein level, by transient expression in cell culture, as well as in the context of cell culture infection. To address these questions under BSL-2 conditions, the replication of furin-primed and unprimed recombinant vesicular stomatitis virus (VSV) particles pseudotyped with EBOV GP (rVSV∆G EBOV GP) was analyzed in the presence and absence of host cell protease inhibitors in Huh-7 and Vero cells. Moreover, the proteolytic processing of EBOV GP wild-type (wt) and EBOV GP_AGTAA furin cleavage site mutant was investigated on protein level in co-expression experiments with the human trypsin-like type II transmembrane serine protease 2 (TMPRSS2). The functionality of further generated EBOV GP_AGTAA cleavage site mutants was analyzed with transcription and replication-competent virus-like particles (trVLPs).

## 2 Results

### 2.1 Trypsin-like proteases support cleavage of EBOV preGP upon entry in the absence of furin and cathepsins

The EBOV surface fusion protein GP possesses the multibasic RRTRR sequence at position 497 to 501, with basic aa in P1, P2 and P4 being a classical recognition motif for the proprotein convertase furin (Molloy et al., 1992; Vey et al., 1992), which is processed after R501 (Fig. 1A) (Volchkov et al., 1998; Wool-Lewis and Bates, 1999). In order to investigate the role of the initial GP priming by furin upon virus entry a recombinant VSV bearing EBOV GP instead of VSV G was treated with the potent substrate analogue furin inhibitor MI-1148 (K_i_ value of 5.5 pM.) to generate rVSV∆G EBOV virus stock with uncleaved preGP (rVSV∆G EBOV preGP). MI-1148 has been described to efficiently inhibit the furin-catalyzed activation and spread of different viruses including H5N1 and H7N1 highly pathogenic avian influenza viruses, respiratory syncytial virus, as well as West-Nile virus and Dengue virus (Hardes et al., 2015; Steinmetzer and Hardes, 2018). Hence, Huh-7 cells were infected with rVSV∆G EBOV GP at a high multiplicity of infection (MOI) of 0.5 for 24 h with or without treatment with 30 µM MI-1148. Western blot analysis of the viral stock preparations confirmed that incubation of cells with MI-1148 prevented GP cleavage leading to the incorporation of the 160 kDa unprimed GP (preGP) into rVSV∆G EBOV progeny particles. In contrast, cleaved GP_1/2_ was detected in virus particles released from untreated cells (Fig. 1B).

For subsequent comparative infections, untreated VeroE6 or Huh-7 cells were inoculated with the rVSV∆G EBOV virus stocks containing cleaved GP_1/2_ or uncleaved preGP at a low MOI of 0.005 for 72 h. The viral growth kinetics showed an initial delay in viral replication for the unprimed rVSV∆G EBOV preGP stock in both VeroE6 and Huh-7 cells, which was overcome in the later stages of infection, implicating a disadvantage resulting from the missing furin cleavage upon entry that was compensated during the infection (Fig. 1C, left panels). To further investigate the proteolytic activation of unprimed rVSV∆G EBOV preGP in VeroE6 and Huh-7 cells, infections were performed under single and combined furin (MI-1148), endosomal cathepsins (E64d) and trypsin-like protease (BAPA) inhibitor treatment for 72 h (Fig. 1C, middle and right panels).

The replication of rVSV∆G EBOV preGP in VeroE6 cells was sensitive to both MI-1148 and E64d, respectively, reducing viral titers to comparable levels. Interestingly, combined treatment with MI-1148 and E64d led to a slight additional titer reduction, but no complete abrogation of virus replication was observed. This indicates that other cellular proteases could be involved in GP activation. In order to investigate whether trypsin-like proteases are able to compensate the lack of furin and endosomal cathepsins, VeroE6 cells were treated with the broad trypsin-like serine protease inhibitor BAPA (Sielaff et al., 2011). Single BAPA treatment without inhibition of furin and cathepsins had no significant effect on rVSV∆G EBOV preGP replication. In contrast, a combined treatment with MI-1148, E64d and BAPA was able to further reduce viral titers. Hence, indicating that the presence of an unknown trypsin-like protease helps to overcome the initial growth disadvantage caused by absent furin-catalyzed priming (Fig. 1C, upper panel).

Interestingly, the results differed significantly in Huh-7 cells infected with unprimed rVSV∆G EBOV preGP compared to VeroE6 cells. Under the single MI-1148 treatment no titer reduction was observed. In contrast, incubation with E64d had a strong inhibitory effect, especially at early time points p.i., displaying a complete initial inhibition of virus replication with a slight increase of viral titers after 48 h p.i. Moreover, incubation of combined MI-1148 and E64d was not able to further reduce the viral titers (Fig. 1C, lower panel). The data indicate a crucial role of endosomal cathepsins for the entry and replication of rVSV∆G EBOV preGP in Huh-7 cells with cathepsins compensating for the lack of furin during initial GP processing. Even though E64d strongly inhibited rVSV∆G EBOV preGP replication in Huh-7 cells, the effect of additional trypsin-like protease inhibitor BAPA was investigated. Single treatment with BAPA had no effect on rVSV∆G EBOV preGP replication similar to the observations made in VeroE6 cells. Nevertheless, combined treatment of MI-1148, E64d and BAPA led to complete abrogation of virus replication over 72 h in Huh-7 cells further indicating that trypsin-like protease(s) are able to process EBOV GP to some extent in the absence of furin and endosomal cathepsins.

In order to address this observation, further comparative growth kinetics of the furin-primed and unprimed rVSV∆G EBOV stock were performed in Vero cells stably expressing TMPRSS2 (Vero-TMPRSS2 cells) (Hoffmann et al., 2020). Intriguingly, viral replication was very efficient in the presence of TMPRSS2 as a 10-fold reduced inoculation dose for both rVSV∆G EBOV stocks had to be used in Vero-TMPRSS2 cells in order to achieve similar growth kinetics compared to VeroE6 and Huh-7 cells. Moreover, the initial disadvantage in rVSV∆G EBOV preGP replication observed in VeroE6 cells (Fig. 1C, upper left panel) completely vanished in Vero-TMPRSS2 cells (Fig. 1D) demonstrating that trypsin-like host cell proteases, particularly TMPRSS2, can compensate for the lack of furin priming of EBOV GP upon virus entry.

To confirm that TMPRSS2 can cleave wild-type (wt) EBOV GP a V5-tagged EBOV GP_wt, which allows both precursor preGP and GP_2_ to be detected by immunoblotting, was co-expressed with the human TMPRSS2 in the presence and absence of the furin inhibitor MI-1148 in HeLa cells for 24 h. Subsequently, the cells were harvested and GP cleavage was analyzed by SDS-PAGE and western blotting using a V5-specific antibody. Expression of the EBOV GP_wt under the treatment with MI-1148 abolished furin cleavage with a clear shift from processed GP_2_ to the uncleaved preGP when compared with non-inhibited expression (Fig. 1E, lane 1 and 2). Furin cleavage of EBOV GP_wt was highly efficient without the TMPRSS2 co-expression having an additional effect. Nevertheless, cleavage of preGP by TMPRSS2 was observed in the absence of furin activity (Fig. 1E, lane 3 and 4). A 110 kDa form of GP representing the precursor present in the endoplasmic reticulum (preGP_ER_) was consistently detected as described previously (Volchkov et al., 1995; Volchkov et al., 1998) and was identified as such by deglycosylation using endoglycosidase H (EndoH, data not shown).

### 2.2 Furin cleavage site mutant EBOV GP_AGTAA is cleaved by TMPRSS2

**Fig. 2:**
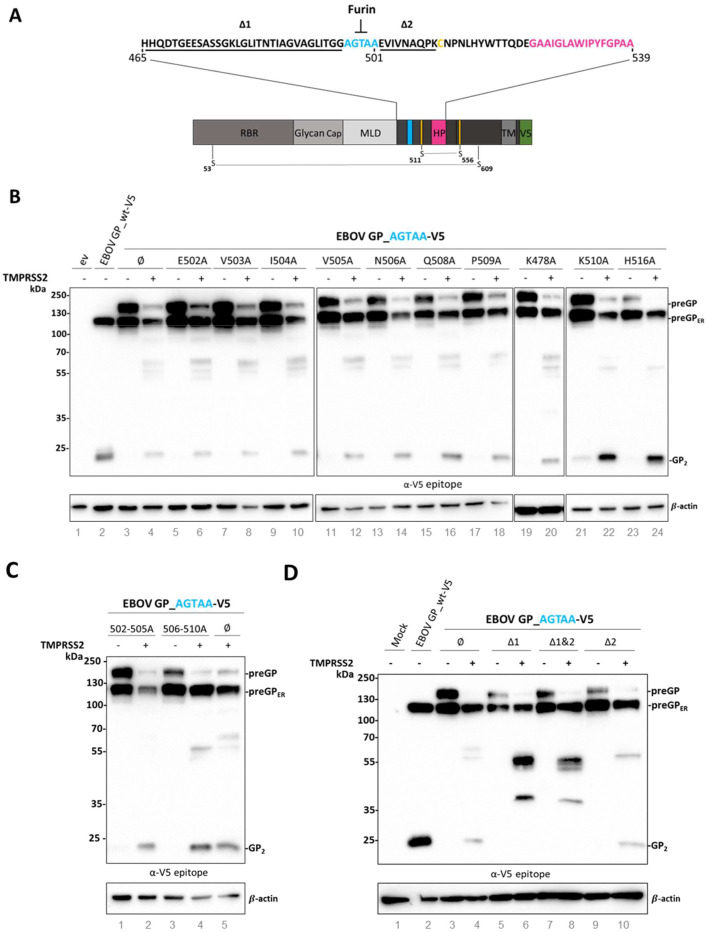
Identification of trypsin-like protease cleavage site in EBOV GP_AGTAA mutant. A) Schematic illustration of C-terminally V5-tagged EBOV GP. The region of interest from aa 465 to 539 is enlarged, comprising the mutated furin cleavage site with the AGTAA sequence (cyan), the aa forming the hydrophobic patch (magenta) and the C511 (yellow) involved in the disulfide bridge to C556 forming the internal fusion loop. Underlined aa indicate deletions from aa 465 to 496 (∆1) and aa 502 to 510 (∆2). B) Proteolytic cleavage of EBOV GP_AGTAA mutants by human TMPRSS2. HeLa cells were co-transfected with plasmids encoding for EBOV GP_wt, EBOV GP_AGTAA without aa exchange (∅) or single alanine scanning mutants from aa position 502 to 509 as well as basic aa K478, K510 and H516 with human TMPRSS2 plasmid for 24 h. C) EBOV GP_AGTAA without aa exchange (∅) or multiple alanine mutants from aa position 502–505 and 506–510 were co-expressed with human TMPRSS2 or empty vector (ev) in HeLa cells for 24 h. D) HeLa cells were co-transfected with plasmids encoding for EBOV GP_AGTAA, EBOV GP_AGTAA∆1, EBOV GP_AGTAA∆2 and EBOV GP_AGTAA∆1&2 and human TMPRSS2 for 24 h. The cells were then harvested, lysed and the samples subjected to a 10 % SDS-PAGE and immunoblotting. EBOV GP and its cleavage forms were detected with a V5-specific antibody. ß-Actin staining was used as a loading control. preGP: uncleaved precursor; preGP_ER_: uncleaved precursor in endoplasmic reticulum (ER). Western blots shown are representative immunoblots of three independent experiments (*n* = 3). Lanes are identified by gray numbers.

The stronger suppression of rVSV∆G EBOV preGP replication by addition of BAPA in VeroE6 and Huh-7 cells as well as its enhanced replication in Vero-TMPRSS2 cells (Fig. 1C & D) hinted a possible proteolytic activation of unprimed EBOV GP by trypsin-like serine proteases including TMPRSS2. To further investigate proteolytic cleavage of GP by TMPRSS2, we exploited a previously described furin cleavage site mutant in which the multibasic RRTRR motif of EBOV GP_wt was substituted by an "uncleavable" non-basic AGTAA motif (Fig. 2A) (Neumann et al., 2007). V5-tagged EBOV GP_AGTAA was co-expressed with human TMPRSS2 in HeLa cells as described above and GP cleavage was analyzed by SDS-PAGE and western blotting. Western blot analysis revealed that EBOV GP_AGTAA was enzymatically cleaved upon co-expression with TMPRSS2 leading to an approximately 20 kDa GP product. The cleavage product had a similar molecular weight as observed for EBOV GP_wt processed by endogenous furin (Fig. 2B, lane 2–4). This was an unexpected finding since trypsin-like proteases, including TMPRSS2, prefer basic aa in the P1 position of their substrates and no basic residues were present in the mutated AGTAA motif or close to it. Hence, to identify the alternative TMPRSS2 cleavage site within the EBOV GP_AGTAA a systematic alanine (A) scan near the mutated AGTAA furin cleavage site from aa position 502 to 509 (EVIVNAQP) was performed (Fig. 2A). Single alanine substitution at position E502, V503, I504, V505, N506, Q508 or P509 did neither alter protein expression nor change the cleavability of EBOV GP_AGTAA by TMPRSS2Fig. 2B, lane 5–18). Therefore, in a next alanine scanning approach the three most adjacent basic aa K478, K510 and H516 were mutated. However, EBOV GP_AGTAA K478A, K510A and H516A mutants were still processed, indicating that none of these basic aa was involved in the cleavage of EBOV GP_AGTAA upon TMPRSS2 co-expression (Fig. 2B, lane 19–24). As the single aa substitutions were not able to inhibit TMPRSS2 cleavage, an unconventional stretch of aa could be used as a substrate for TMPRSS2. Thus, further mutants with an alanine exchange from position 502 to 505 (502–505A) and 506 to 510 (506–510A) were generated. Nevertheless, both EBOV GP_AGTAA mutants were cleaved upon co-expression with TMPRSS2 in HeLa cells (Fig. 2C, lane 1–4). Thus, identification of the exact cleavage site within the EBOV GP_AGTAA with specific single or multiple alanine exchanges in close proximity to the mutated furin cleavage site motif was not successful. Next, broad aa deletions were made upstream from the end of the mucin-like domain (MLD) to the start of the AGTAA motif (aa 465 to 496), designated Δ1, and downstream from the end of the AGTAA motif to aa 510 (Δ2) together with a variant lacking both stretches (Δ1&2) (Fig. 2A). Despite the deletion of 9, 32 or 41 aa, all preGP forms of the three EBOV GP_AGTAA deletion mutants were similarly expressed upon transient expression with a slight decrease in the detection of uncleaved preGP compared to the EBOV GP_AGTAA lacking further mutations (Fig. 2D, lane 3, 5, 7 and 9). EBOV GP_AGTAA Δ2 was still processed by TMPRSS2 and the 20 kDa GP cleavage product was detected, indicating that the 9 aa after the AGTAA motif are not critical in the TMPRSS2-mediated processing (Fig. 2D, lane 10). In contrast, EBOV GP_AGTAAΔ1 and EBOV GP_AGTAAΔ1&2 mutants were cleaved into higher molecular weight products but not into 20 kDa GP by TMPRSS2 (Fig. 2D, lane 6 and 8). These data suggest, that either a stretch of unusual aa is recognized by TMPRSS2 within the area from position 465 to 496, or the deletion of these 32 aa leads to a conformational change in the EBOV GP protein that sterically hinders TMPRSS2-induced processing leading to a cleavage product with similar size as GP_2_. The exact TMPRSS2 cleavage site in EBOV GP_AGTAA, however, remains unknown.

### 2.3 Functional analysis of EBOV GP_AGTAA Δ1&2 bearing trVLPs

The EBOV GP_AGTAA∆1 and ∆1&2 deletion mutants in comparison to the previously described EBOV GP_AGTAA were no longer processed by furin and TMPRSS2 into GP_2_ in co-expression experiments, representing a first non-cleavable mutant. Hence, we wanted to assess whether these EBOV GP variants are functional and support membrane fusion and virus entry.

**Fig. 3:**
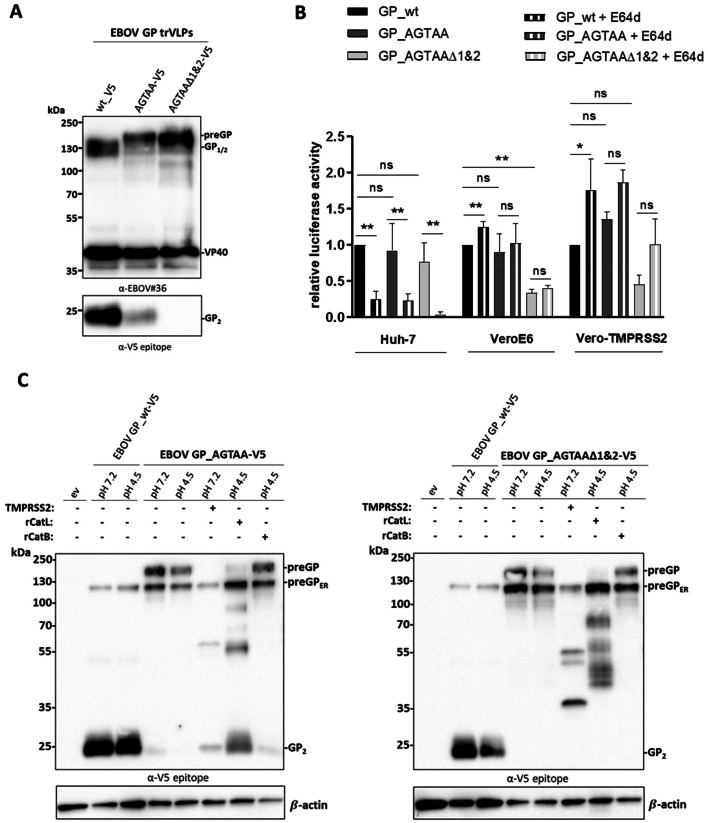
Functional analysis of EBOV GP_AGTAA and GP_AGTAA∆1&2 in trVLP assay reveals an alternative processing by endosomal cathepsins in Huh-7 cells. A) Transcription and replication-competent virus-like particles (trVLPs) were generated in HEK293 cells. After 72 h cellular supernatants were harvested and trVLPs were concentrated on a sucrose cushion by ultracentrifugation. The trVLP pellets were resuspended in PBS, samples were subjected to a 10 % SDS-PAGE and analyzed by western blot. EBOV proteins were detected by an EBOV-specific serum while for detection of GP_2_ a V5 epitope antibody was used. B) Entry and reporter genome replication of EBOV GP trVLPs. Huh-7, VeroE6 and Vero-TMPRSS2 cells were incubated with or without E64d (20 µM) for 1 h and subsequently inoculated with trVLPs bearing EBOV GP_wt, GP_AGTAA or GP_AGTAA∆1&2 for 72 h. Afterward, the cells were harvested and lysed for 30 min on ice. Renilla luciferase reporter gene signal was normalized to firefly luciferase activity and GP_wt activity was set to 1. Data shown are mean values (+SD) of three to six (*n* = 3-6) independent experiments. For statistical group analysis of differences between untreated EBOV GP variants, a Kruskal-Wallis one-way ANOVA on ranks with subsequent Dunn´s multiple comparison test was performed. Further comparison of treated and untreated samples was statistically analyzed by Mann-Whitney test. Statistically significant p values are represented as followed: ≤ 0.05 (*) and ≤ 0.01 (**), whereas p values of >0.05 were considered not significant (ns). C) Alternative cleavage of overexpressed EBOV GP_AGTAA and GP_AGTAA∆1&2 by recombinant cathepsin B and L (rCatB and rCatL) in HeLa cells. HeLa cells were co-transfected with plasmids encoding for EBOV GP_AGTAA or EBOV GP_AGTAA∆1&2 and human TMPRSS2 or empty vector (ev) for 24 h. Subsequently, the cells were harvested, and cellular pellets were incubated with 25 µg/ml of rCatB or rCatL at pH 4.5 for 20 min at 37 °C. Cells were lysed, and the samples subjected to a 10 % SDS-PAGE and immunoblotting. EBOV GP cleavage products were detected using a V5-specific antibody. ß-actin was used as a loading control. Western blots shown are representative immunoblots of four independent experiments (*n* = 4).

For this purpose, we generated transcription and replication-competent virus-like particles (trVLPs) containing a luciferase encoding monocistronic mini genome bearing EBOV GP_wt or mutated GP_AGTAA and GP_AGTAAΔ1&2 at the surface. The trVLPs were produced in HEK293 cells as described in (Hoenen et al., 2006), which express furin but not TMPRSS2 and are highly susceptible to transfection. Subsequent western blot analysis of the concentrated trVLPs using an EBOV-specific serum showed equal expression levels of the matrix protein VP40 in all three trVLP preparations with comparable levels of incorporated GP1/2 for the EBOV GP_wt and preGP for EBOV GP_AGTAA and GP_AGTAAΔ1&2, respectively (Fig. 3A). Detection of C-terminal cleavage products with an antibody directed against the C-terminal V5 epitope-tag displayed a faint GP2 band for EBOV GP_AGTAA with a similar molecular weight of 20 kDa as the furin-cleaved EBOV GP_wt again indicating an alternative processing of EBOV GP_AGTAA during trVLP generation in HEK293 cells. In contrast, no 20 kDa GP cleavage product could be observed in trVLPs bearing the EBOV GP_AGTAAΔ1&2 deletion mutant (Fig. 3A).

For the trVLP infection Huh-7, VeroE6 or Vero-TMPRSS2 cells were pre-transfected with plasmids encoding VP30, VP35, NP and L as well as a pGL4 firefly luciferase plasmid for normalization 24 h prior to infection. The cells were then treated with the endosomal cathepsin inhibitor E64d or remained untreated for 1 h. Subsequently, cells were inoculated with the respective trVLPs and incubated for 72 h. Cell pellets were harvested, lysed and luciferase activity was measured. In infected Huh-7 and Vero cells, there was no decrease in relative luciferase activity of EBOV GP_AGTAA-bearing trVLPs compared to the EBOV GP_wt showing that here small amounts of cleaved GP are sufficient for membrane fusion (Fig. 3B). In contrast, in VeroE6 and Vero-TMPRSS2 cells a significant reduction in reporter gene activity of 67 % and 55 %, respectively, was detected for trVLP carrying EBOV GP_AGTAAΔ1&2 compared to EBOV GP_wt and EBOV GP_AGTAA. Hence, missing proteolytic processing into the fusion-competent 20 kDa form upon entry seemed to hamper entry and genome release in VeroE6 and Vero-TMPRSS2 cells. Interestingly, this was not the case for Huh-7 cells, here the entry of EBOV GP_AGTAAΔ1&2 trVLPs was only slightly reduced compared to EBOV GP_wt, suggesting sufficient proteolytic activation during virus entry in Huh-7 cells. Probable candidates for GP processing into GP_2_ upon trVLP entry were endosomal CatB and CatL, which are known to trim the GP_1_ subunit for subsequent NPC1 receptor binding during the viral entry (Dube et al., 2009; Chandran et al., 2005; Schornberg et al., 2006; Kaletsky et al., 2007; Brecher et al., 2012). Surprisingly, the treatment of VeroE6 with 20 µM E64d did not lead to a reduction but rather to a slight increase of the reporter gene activity for all three trVLP variants (Fig. 3B). This observation was even more pronounced in Vero-TMPRSS2 cells. Here, EBOV GP AGTAAΔ1&2 trVLPs were also able to enter the cells, however, more efficiently when the endosomal cathepsins were inhibited. This leads to the conclusion that trimming by CatB and CatL was not necessary or even a disadvantage for the EBOV entry into Vero cells. Interestingly, a different outcome was observed for trVLP infections in Huh-7 cell. Inhibition of CatB and CatL significantly inhibited EBOV GP_wt, GP_AGTAA and GP_AGTAAΔ1&2 trVLP entry and reporter gene activity, indicating that endosomal cathepsins are crucial for the entry of all three EBOV GP variants in Huh-7 cells. These findings were also in line with the observed inhibitory effect of E64d on the replication of rVSV∆G EBOV preGP in this cell line (Fig. 1C).

To examine whether the high sensitivity of trVLP entry into Huh-7 cells to E64d treatment is due to a possible cleavage of the EBOV mutants GP_AGTAA and AGTAAΔ1&2 by CatB and CatL, HeLa cells were transfected with the respective GP-expressing plasmid. Co-expression of TMPRSS2 served as a processing control. Cells were harvested after 24 h incubation and treated with recombinant cathepsins (rCatB and rCatL) at low pH conditions. Subsequently, the cell lysates were subjected to SDS-PAGE and western blot analysis using a V5-specific antibody. Western blot analysis revealed that EBOV GP_AGTAA was efficiently processed into the 20 kDa fusion competent GP_2_ by rCatL but not rCatB, showing that this "non-cleavable" variant can be cleaved by both TMPRSS2 and by CatL (Fig. 3C, left panel). In contrast, no 20 kDa GP product was detected upon treatment of EBOV GP_ AGTAAΔ1&2 expressing cells with rCatB and rCatL (Fig. 3C, right panel). However, TMPRSS2 and CatL processed EBOV GP_AGTAAΔ1&2 into higher molecular weight products that seemed to be fusion active as they led to an efficient reporter genome release in Huh-7 and E64d-treated Vero-TMPRSS2 cells, respectively (Fig. 3B). Taken together EBOV GP_ AGTAAΔ1&2 enables a functional entry into Huh-7 cells in a cathepsin l-dependent manner while interestingly inhibition of endosomal cathepsins supports entry of this GP variant into TMPRSS2-expressing Vero cells. Thus, the ability of the EBOV GP cleavage mutants to enter the cell and to release their genome to a sufficient extent appears to be highly dependent on the protease repertoire of the respective cell line. Furthermore, our data show that proteolytic activation of EBOV GP offers enormous flexibility and ample scope for various proteases (Fig. 5).

### 2.4 TMPRSS2 is expressed in late and recycling endosomes

**Fig. 4:**
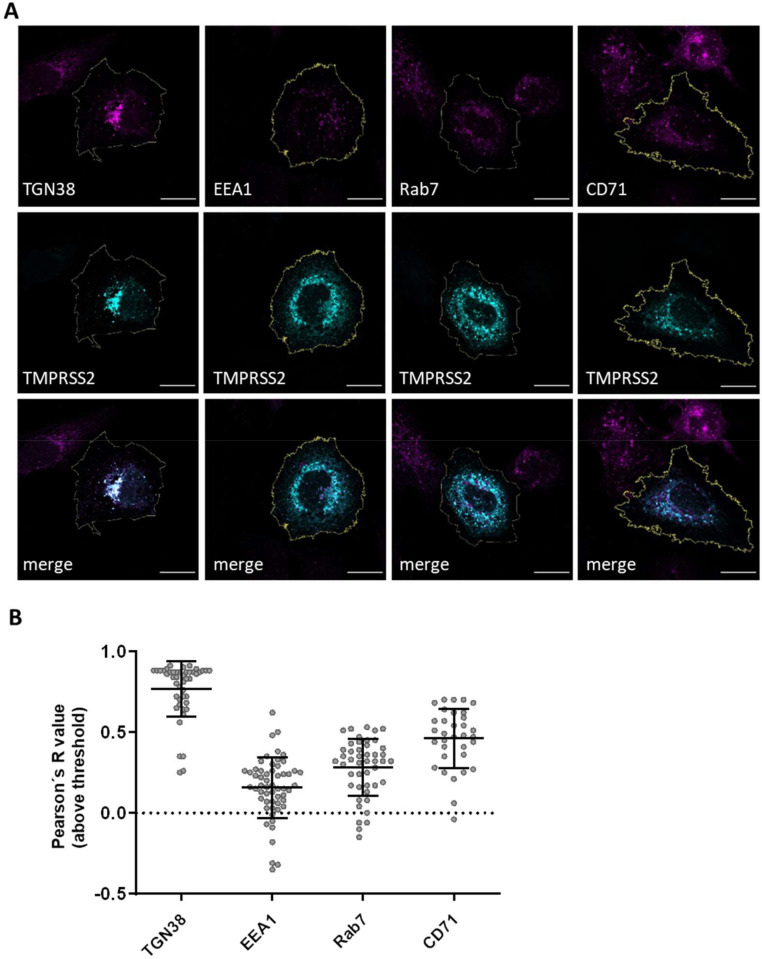
Subcellular localization of transiently expressed TMPRSS2 in Huh-7 cells. A) Co-localization of transiently expressed human TMPRSS2 with cellular compartment markers. Huh-7 cells were transfected with pCAGGS-TMPRSS2-FLAG for 24 h. The cells were fixed, permeabilized and stained using a FLAG-specific antibody and an Alexa-488-coupled secondary antibody. The subcellular compartments were co-stained with specific antibodies against EEA1 (early endosome), Rab 7 (late endosome), TGN38 (TGN), and CD71 (recycling endosome) and an Alexa-568-conjugated secondary antibody. The images shown are representative immunofluorescence images displayed in the red and green channel, as well as a merge. The cellular membrane border (yellow) was defined as the region of interest (ROI), which was used for co-localization analysis (Coloc2, Fiji). B) Quantification of co-localization with Pearson´s correlation coefficient of TMPRSS2-FLAG with compartment marker proteins. Data shown are mean values (±SD) of 33 to 56 analyzed cells from three to five independent experiments (*n* = 3–5).

**Fig. 5:**
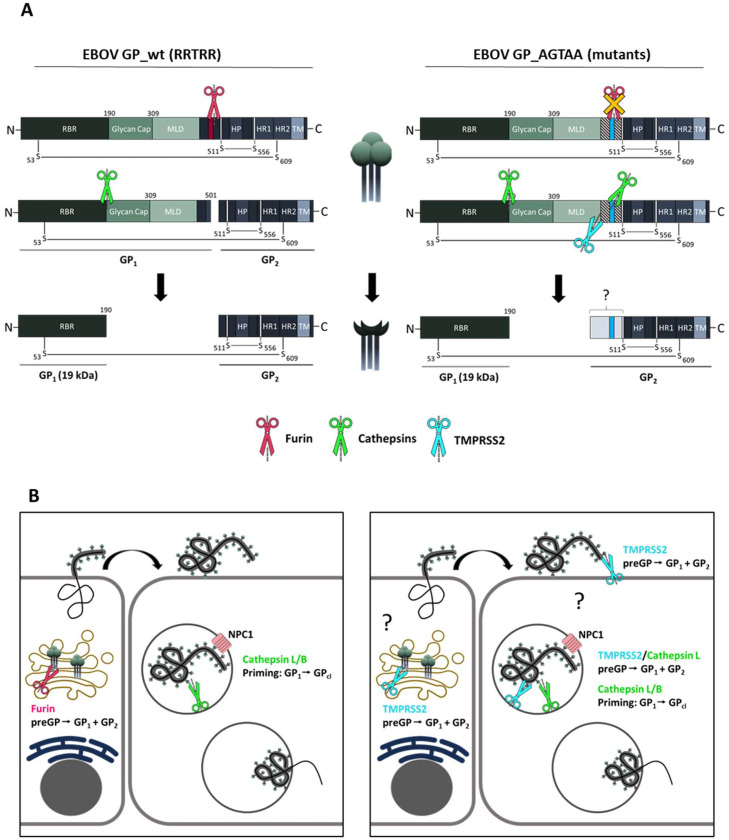
Alternative processing of EBOV GP_AGTAA furin cleavage mutants. A) Schematic illustration of proteolytic activation of EBOV GP_wt by furin and endosomal cathepsins, leading to a fusion competent GP_2_ and GP_1_ with exposed receptor binding region (RBR). Abrogated furin cleavage observed for EBOV GP_AGTAA and EBOV GP_AGTAA∆1&2 (deletions are indicated as hatched areas) is substituted by other host cell proteases, including trypsin-like serine proteases as TMPRSS2 and the endosomal cysteine protease CatL. Processing of GP_AGTAA mutants is carried out at so far uncharted positions resulting in cleavage products with possible higher molecular weights, which remain fusion competent. B) EBOV GP activation model during viral replication cycle. The preGP of EBOV GP_wt is cleaved by furin within the TGN along the secretory pathway into GP_1_ and GP_2_ and subsequently incorporated into budding EBOV particles. Upon entry in new target cells GP_1_ is trimmed by endosomal cathepsins (GP_cl_) leading to NPC1 binding and fusion within the late endosomes. The preGP of EBOV GP_AGTAA furin cleavage mutants can no longer be processed by furin. However, trypsin-like proteases, like TMPRSS2, and endosomal CatL might be able to compensate for lack of furin cleavage leading to fusion competent GP_2_ and free GP_1_ that is further trimmed by endosomal cathepsins for NPC1 binding and subsequent fusion.

TMPRSS2 has been demonstrated to be the major activating protease of several respiratory viruses in human airway cells and in mouse models (Limburg et al., 2019; Li et al., 2021; Iwata-Yoshikawa et al., 2019; Park et al., 2016). Here, we found that TMPRSS2 is also able to cleave EBOV GP_wt as well as the EBOV-GP_AGTAA mutant. TMPRSS2 has been shown to be localized both in the trans-Golgi network (TGN) and at the plasma membrane (Böttcher-Friebertshäuser et al., 2013). Hence, cleavage of EBOV GP by TMPRSS2 could occur in the TGN in the same way as cleavage by furin or at the plasma membrane during entry, as has been described for cleavage of influenza A virus hemagglutinin and coronavirus spike, respectively (Fig. 5B). However, efficient entry of recombinant VSV bearing uncleaved EBOV preGP and trVLP entry under E64d treatment into Vero-TMPRSS2 cells (Fig. 1D and 3 B) indicated that TMPRSS2 is able to support EBOV GP cleavage at the stage of entry during trafficking trough early and late endosomes as well (Saeed et al., 2010; Spence et al., 2016; Wang et al., 2016). We therefore examined whether TMPRSS2 is expressed in endosomal compartments. Huh-7 cells were transiently transfected with a plasmid encoding TMPRSS2 with C-terminal FLAG epitope. At 24 h post transfection, the cells were fixed, permeabilized and stained with a FLAG-specific antibody as well as antibodies specific for early endosomal antigen-1 (EEA1, early endosome), Rab 7 (late endosome), the transferrin receptor CD71 (recycling endosome) or TGN38 (TGN) (Fig. 4A). The Pearson's correlation coefficient, which gives a measure of the linear correlation between two fluorescence signals, was calculated, and is plotted in Fig. 4B. As described previously TMPRSS2 prominently co-localized with TGN38 (mean R value of 0.76). Further, an increasing co-localization of TMPRSS2 within the endosomal compartments was observed, showing only low correlation with EEA1 (mean R value of 0.15) and an increasing partial co-localization with Rab7 (mean R value of 0.28) and CD71 (mean R value of 0.47). The data indicate that TMPRSS2 is localized to some extend in late and recycling endosome and may support EBOV GP cleavage upon virus entry in the late endosome.

## 3 Discussion

The role of EBOV GP processing in virus infectivity and the proteases responsible for cleavage has been and still is a matter of debate*. In vitro* studies revealed a sequential activation of EBOV GP during virus replication by furin during GP synthesis with a subsequent trimming by endosomal cathepsins upon virus entry (Volchkov et al., 1998; Wool-Lewis and Bates, 1999; Chandran et al., 2005; Schornberg et al., 2006; Kaletsky et al., 2007; Brecher et al., 2012) and a further involvement of CatB in following fusion triggering events (Odongo et al., 2023). Nevertheless, in subsequent *in vitro* and *in vivo* studies, no significant decrease in infectivity and pathogenicity of EBOV was observed in the absence of furin or CatB and CatL cleavage (Wool-Lewis and Bates, 1999; Neumann et al., 2002; Neumann et al., 2007; Marzi et al., 2012).

Here, we show that proteolytic activation of EBOV GP is a highly complex process that may involve trypsin-like host cell proteases in addition to the previously identified activating proteases, furin and endosomal cathepsins. Recombinant VSV particles containing uncleaved EBOV preGP on the virus surface (rVSVΔG EBOV preGP) were generated by using the peptide-mimetic furin inhibitor MI-1148. Thus, the proteolytic cleavage of EBOV GP_wt by furin was not a prerequisite for GP incorporation into the viral membrane, as has been previously observed (Wool-Lewis and Bates, 1999; Neumann et al., 2002). Comparative growth kinetics of rVSVΔG bearing cleaved EBOV GP1/2 or uncleaved EBOV preGP in VeroE6 and Huh-7 cells revealed an initial replication disadvantage for the unprimed preGP, which, however, was overcome after 48 h. This difference in the respective replication curves was similar to the effect observed in VeroE6 infection studies by Neumann et al. with recombinant EBOV viruses containing a non-basic AGTAA sequence (Neumann et al., 2002). This suggests that there might be a furin-independent processing of EBOV GP_AGTAA upon entry into the cell.

The treatment of VeroE6 cells with inhibitors either against furin or endosomal cathepsins led to a reduction of viral titers in rVSVΔG EBOV preGP infected cells, but was not able to abrogate viral replication in line with previous studies (Neumann et al., 2002; Neumann et al., 2007; Marzi et al., 2012). Nevertheless, we were able to show that combined treatment with MI-1148 and E64d had a synergistic effect on the replication of rVSVΔG EBOV preGP and strongly reduced virus titers in VeroE6 cells. Interestingly, only the addition of the broad trypsin-like serine protease inhibitor BAPA in combination with furin and cathepsin inhibitors was able to strongly suppress the replication of unprimed rVSVΔG EBOV preGP in VeroE6 cells and completely abolished viral titers in Huh-7 cells, demonstrating that trypsin-like proteases can partially substitute for the proteolytic processing in the absence of the previously known EBOV GP-activating enzymes. This was further supported by infection studies in Vero-TMPRSS2 cells. Viral replication was very efficient, as a 10-fold lower infection dose had to be used to obtain comparable virus titers in contrast to VeroE6 cells. Moreover, the initial growth disadvantage of rVSVΔG with unprimed EBOV preGP in VeroE6 cells was not observed in Vero-TMPRSS2 cells, strongly suggesting that trypsin-like proteases, particularly TMPRSS2, are able to activate EBOV GP during virus entry.

Infection studies with trVLPs bearing either EBOV GP_wt, GP_AGTAA or GP_AGTAAΔ1&2 in Huh-7 cells revealed functional fusion capacity for all three GPs, but only after proteolytic activation upon entry. Treatment with E64d resulted in a significantly lower reporter gene expression for all three EBOV GP variants in Huh-7 cells, indicating that processing of GP is carried out predominantly by endosomal cathepsins in these cells. In line with this, viral replication of rVSV∆G ZEBOV preGP in Huh-7 cells was strongly impaired under the treatment with E64d, while MI-1148 had no effect. We were able to show that CatL, but not CatB, is able to process the EBOV GP_AGTAA mutant to the 20 kDa GP fragment, revealing an even more complex, previously unknown, possible GP_1/2_ trimming by CatL during viral entry.

In contrast to Huh-7 cells, infection of VeroE6 cells with trVLPs bearing EBOV GP_AGTAAΔ1&2 showed a significant decrease in reporter activity compared to the EBOV GP_wt indicating that endosomal CatB and CatL are unable to efficiently activate this mutant in these cells. Moreover, treatment of VeroE6 cells with E64d increased the reporter gene activity for all three EBOV GP variants. These observations indicated a so far unknown additional GP-activating protease(s) within the endosomal compartments with similar cleavage efficiency. The enhanced entry of trVLPs bearing either EBOV GP_wt, GP_AGTAA or GP_AGTAA∆1&2 was even more pronounced in Vero-TMPRSS2 cells. This was rather unexpected and indicated that TMPRSS2 might support a more efficient activation of EBOV GP during entry compared to endosomal cathepsins. One may speculate that CatL and TMPRSS2 compete for EBOV GP cleavage in the endosome, with cathepsins having a greater turnover, but TMPRSS2 being more precise or efficient. Analysis of EBOV GP_AGTAAΔ1&2 processing by CatL revealed several cleavage fragments, probably due to sequential trimming of GP. In contrast, processing of EBOV GP_AGTAAΔ1&2 by TMPRSS2 resulted in one major fragment of approximately 37 kDa (Fig. 3C). It has been shown that fusogenic triggering of EBOV GP occurs predominantly in late endosomes after macropinocytosis and involves a number of steps in addition to GP trimming by CatL/CatB and low pH, which are not yet fully understood (Odongo et al., 2023; Das et al., 2020; Spence et al., 2016). We found that TMPRSS2 co-localizes with the late endosomal marker protein Rab7, suggesting that cleavage of EBOV GP by TMPRSS2 might be possible in this compartment. However, whether TMPRSS2 is involved in GP priming in addition to CatL in late endosomes or cleaves preGP into GP1 and GP2 at earlier stages of the viral life cycle remains to be clarified (Fig. 5). We note that our data are based on overexpression of TMPRSS2 and should be confirmed in future studies for endogenous TMPRSS2.

In sum, we show that proteolytic activation of EBOV GP clearly differed in Huh-7 and VeroE6 cells. Processing in VeroE6 cells appeared to be complex including furin, endosomal cathepsins and trypsin-like serine proteases. In contrast, the protease profile of EBOV GP-activating host cell proteases seemed to be rather narrow in Huh-7 cells with a high dependency on endosomal cathepsins.

The EBOV GP_AGTAA mutant has been described as a GP variant that cannot be cleaved anymore at or close to the furin cleavage site. Hence, replication of recombinant EBOV bearing EBOV GP_AGTAA in Vero cells and in non-human primates was taken as evidence that proteolytic cleavage of EBOV GP is not crucial for its fusion capacity (Neumann et al., 2002; Neumann et al., 2007). Here, we found that both TMPRSS2 and CatL can cleave the EBOV GP_AGTAA mutant into a fusion-competent 20 kDa GP product with similar molecular weight as the EBOV GP_wt cleaved by endogenous furin (Fig. 3C). Additionally, a low level of cleavage of EBOV GP_AGTAA mutant was observed in HEK293 cells by a yet unknown endogenous protease (Fig. 3A). Therefore, the EBOV GP AGTAA mutant does not represent a "non-cleavable" GP. Our data indicate that the EBOV GP_AGTAA was also proteolytically activated by TMPRSS2 and/or CatL in earlier studies in cell culture and nonhuman primates (Neumann et al., 2002; Neumann et al., 2007) and therefore argue against the statement that GP cleavage is dispensable for EBOV replication. Hence, our results support the concept that cleavage of EBOV GP is required for its fusion activity, but also show that EBOV GP exhibits great flexibility in both the cleavage site and the proteases involved.

Our study together with previous studies suggest that the furin cleavage site and adjacent regions appear to be easily accessible so that they can be recognized and cleaved by a variety of host cell proteases, providing flexibility to be functionally processed. The substitution of the EBOV GP_wt RRTRR motif by the AGTYF chymotrypsin recognition sequence still enabled *in vitro* cleavage of EBOV GP (Wool-Lewis and Bates, 1999). Moreover, EBOV GP was shown to be quite insensitive to mutations at the furin cleavage site, as the dibasic RATAR and more interestingly, the monobasic RATAA variants were still processed upon transient expression in HEK293 cells (Wool-Lewis and Bates, 1999).

Here, we demonstrated that even the EBOV GP_AGTAA mutant is cleaved by TMPRSS2 or CatL into a fusion-competent GP_2_ form. The unique structure of the internal fusion loop of GP_2_ might contribute to the vast resistance of EBOV GP to cleavage site mutations and its flexibility in being proteolytically activated. In contrast to many other class I fusion proteins, including the influenza A virus HA with the fusion peptide situated at the free N-terminus of the HA2 subunit after HA cleavage, the hydrophobic patch of EBOV GP_2_ is located at the tip of the internal fusion loop formed by a disulfide bridge between C511 and C556. The interaction of both cysteine residues has been shown to be pivotal for the transduction efficiency of GP-bearing reporter viruses (Jeffers et al., 2002; Gregory et al., 2011). Hence, the formation of the hairpin structure could be allowed even with longer overlaps than the 9 aa resulting from furin cleavage at position 501, as long as the C511 remains intact (Fig. 5A). This could explain the conserved functionality of the EBOV GP_AGTAAΔ1&2 variants in trVLP entry assays. Even though no 20 kDa product was detected in the trVLP purifications, *in vitro* processing of EBOV GP_AGTAAΔ1&2 by TMPRSS2 and CatL revealed higher molecular weight products presumably still fusion competent (Fig. 3C).

TMPRSS2 belongs to the family of type II transmembrane serine proteases (TTSPs) and preferentially cleaves substrates with a single basic aa, including R or K at the P1 position (Böttcher et al., 2006; Lucas et al., 2014). Interestingly, the EBOV GP possesses no basic aa in close proximity to the AGTAA motif. The most adjacent basic aa found were the two lysines K478 and K510, as well as the histidine H516 (Fig. 2A). Mutation of the basic aa to alanine did not alter the recognition and processing upon co-expression with TMPRSS2, implicating a more unusual substrate sequence recognized by TMPRSS2 within the EBOV GP_AGTAA (Fig. 2B). However, as the molecular weight of the furin cleavage site mutant processed GP2 by TMPRSS2 did not significantly differ from the furin-cleaved EBOV GP_wt, the proteolytic cleavage had to occur at or near the exchanged AGTAA sequence. Nevertheless, the TMPRSS2 cleavage site could not be identified with a systematic alanine scan of the EVIVNAQP sequence from position 502 to 509, including multiple aa stretches. Finally, deletion of 32 aa upstream (Δ1) alone and in combination with a deletion of 9 aa downstream the furin cleavage site resulted in EBOV GP mutants (Δ1 and Δ1&2) that are not cleaved into 20 kDa GP by TMPRSS2 anymore, suggesting that TMPRSS2-mediated processing most likely takes place upstream of the furin cleavage site (Fig. 2D). At this point, it cannot be completely ruled out that the cleavage is indirectly performed by a protease that is activated by TMPRSS2.

Combined treatment of VeroE6 cells using MI-1148, E64d and BAPA strongly suppressed rVSV∆G ZEBOV preGP titers but did not completely inhibit virus replication. This was either due to the decreasing effect of the inhibitors over time or to the activity of other proteases insensitive to the used three protease inhibitors. Although we have no evidence, it might be possible that also metallo- or aspartyl proteases could be involved in the EBOV GP processing in VeroE6 cells. The metalloprotease a disintegrin and metalloprotease 17 (ADAM17/TACE) has been shown to shed the ectodomain of EBOV GP (shedGP) from the cell surface after cleavage at position D637 close to the transmembrane domain (Dolnik et al., 2004). Thus, in general interaction and processing of EBOV GP by metalloproteases has been shown. However, the extent to which metalloproteases or lysosomal aspartyl proteases, such as cathepsin D, are also involved in proteolytic activation of EBOV GP remains to be investigated.

In conclusion, our data show that EBOV GP must be proteolytically activated to support virus entry, but has enormous flexibility in terms of proteases and the exact cleavage sites (Fig. 5). We report a so far not described GP_1/2_ processing by endosomal CatL beyond the already described GP_1_ trimming upon viral entry. Moreover, we show that TMPRSS2 and other trypsin-like proteases can support EBOV GP activation in addition to furin and endosomal cathepsins. This certain independence from a specific GP-activating host cell protease could contribute to the broad organ tropism of the EBOV. Our data also support the hypothesis of previous studies that the inhibition of GP cleavage in EBOV infection, unlike many other viruses, would require a combination of protease inhibitors (Marzi et al., 2012; Ströher et al., 2007).

## 4 Material and methods

### 4.1 Cells and viruses

HeLa (CVCL_0030, RRID), HEK293 (CVCL_6642, RRID), Huh-7 (CVCL_0336, RRID), VeroE6 (CVCL_0574, RRID) and Vero-TMPRSS2 (kindly provided by Stefan Pöhlmann, German Primate Center, Göttingen (Germany) (35)) cells were maintained at 37 °C and 5 % CO_2_ in DMEM supplemented with 10 % fetal calf serum (FCS), antibiotics (penicillin and streptomycin) and glutamine (10 % DMEM+++).

Recombinant VSVΔG expressing the surface glycoprotein (GP) of EBOV Makona C7 isolate (rVSVΔG EBOV GP) were cloned as previously described (Garbutt et al., 2004). The original full-length plasmid was provided by J. Rose (Yale University School of Medicine). Virus rescue was performed using a modified protocol. Briefly, BHK-21 and Huh7 cells co-cultures were infected with MVA-T7 at an MOI of 1 and subsequently transfected with support plasmids coding VSV-N, P and L and full length plasmid as previously described (Garbutt et al., 2004). Culture supernatants were collected 48–72 h p.i. and passaged on VeroE6 and Huh7 cells co-culture. After CPE development the supernatants were harvested and filtrated through a 0.2 µm pore filter to remove MVA-T7. The rVSVΔG EBOV GP was propagated on Huh-7 cells in 10 % DMEM+++. For generation of uncleaved rVSVΔG EBOV preGP cells were infected at a high MOI of 0.5 and incubated in 10 % DMEM+++ containing 30 µM of the potent furin inhibitor MI-1148 for 24 h until the virus stock was harvested. Cellular supernatants were collected and cleared of debris by centrifugation at 2500 rpm for 10 min. Virus stock preparations were stored at −80 °C.

### 4.2 Plasmids and enzymes

The pCAGGS plasmid encoding the EBOV GP (Makona C7 isolate, GenBank KJ660347.2) as an 8A editing variant has been described previously (Krähling et al., 2016). The pCAGGS EBOV GP_wt and furin cleavage site mutant pCAGGS EBOV GP_AGTAA containing a C-terminal V5-tag were generated using site-directed mutagenesis PCR. The following pCAGGS EBOV GP_AGTAA-V5 alanine scan and deletion variants were used in this work: K478A, E502A, V503A, I504A, V505A, N506A, Q508A, P509A, K510A, H516A, 502–505A, 506–510A, deletion of aa 465 to 496 (Δ1), deletion of aa 502 to 510 (Δ2) and double deletion variant (Δ1&2). All primer sequences are available on request. For co-transfection experiments transcript variant 2 of human TMPRSS2 in pCAGGS was used (Böttcher et al., 2006).

For the generation of transcription and replication-competent virus-like particles (trVLPs) (Hoenen et al., 2006) pCAGGS plasmids encoding for EBOV NP, VP24, VP30, VP35, VP40, and L (EBOV Mayinga) as well as T7 polymerase were transfected together with the above mentioned pCAGGS plasmids encoding GP_wt or mutants. The monocistronic reporter mini genome 3E5E encoding for renilla luciferase and pGL4 encoding for firefly luciferase were used for entry read-out and normalization.

Recombinant cathepsin B and L (rCatB and rCatL) were purchased from R&D systems (953-CY and 952-CY).

### 4.3 Protease inhibitors

The cysteine protease inhibitor E64d was purchased from Sigma-Aldrich (E8640). The synthetic inhibitors of TMPRSS2 (BAPA) and furin (MI-1148) were synthesized in-house according to previous methods (Hardes et al., 2015; Sielaff et al., 2011). Stock solutions of protease inhibitors were prepared in double distilled water (BAPA and MI-1148) or sterile DMSO (E64d) and stored at −20 °C.

### 4.4 Antibodies

A polyclonal serum against EBOV generated by immunization of goats (α-EBOV#36) was used for the detection of GP and VP40 in western blot analysis. A monoclonal rabbit antibody directed against the C-terminal V5-tag of the EBOV GP constructs was purchased from Novus Biologicals (NB600–381). A monoclonal mouse anti-β actin antibody was purchased from Abcam (ab6276). HRP-conjugated secondary antibodies were purchased from DAKO. Antibodies used for immunofluorescence microscopy were polyclonal rabbit anti-CD71 (D7G9X; Cell Signaling Technology), monoclonal anti-EEA1 (PA1–063A; Thermo Fisher Scientific), monoclonal rabbit anti-Rab7 (ab137029; Abcam), a polyclonal rabbit serum against TGN38 (Institute of Virology, Marburg) and monoclonal mouse anti-FLAG (F1804; Sigma-Aldrich). Alexa-488- or Alexa 568-conjugated secondary antibodies were purchased from Invitrogen or Life Technologies.

### 4.5 Transient expression of EBOV GP_AGTAA variants in HeLa cells

For transient expression of EBOV GP_AGTAA variants 70 % confluent HeLa cells were co-transfected with 0.8 μg of pCAGGS EBOV GP_AGTAA-V5 variants and either 15 ng of empty pCAGGS vector or pCAGGS-TMPRSS2 using Lipofectamine 2000 (Invitrogen) according to the manufacturers protocol for 24 h. Cells were harvested and centrifuged for 5 min at 8.000x*g*. For cleavage of transiently expressed EBOV GP by exogenous proteases, cellular pellets were resuspended in transfection medium (pH 4.5) containing 25 µg/ml rCatB or rCatL and incubated for 20 min at 37 °C. Subsequently, cells were subjected to SDS–PAGE and western blot analysis as described below.

### 4.6 SDS-PAGE and western blot analysis

Cells were harvested with PBS and cellular pellets were lysed in CelLytic M buffer (Sigma-Aldrich) with a protease inhibitor cocktail (P8340; Sigma-Aldrich) for 30 min on ice, resuspended in reducing SDS-PAGE sample buffer, and heated at 95 °C for 10 min. Proteins were subjected to 8 % or 10 % SDS-PAGE, transferred to a polyvinylidene difluoride (PVDF) membrane (Cytiva Amersham), and detected by incubation with primary antibodies (α-V5 and α-EBOV serum) and species-specific peroxidase-conjugated secondary antibodies. Proteins were visualized using the ChemiDoc XRS system with Image Lab software (Bio-Rad).

### 4.7 Infection of cells and multicycle virus replication in the presence and absence of protease inhibitors

Infection experiments with rVSVΔG EBOV GP_1/2_ or preGP of Huh-7, VeroE6 and Vero-TMPRSS2 cells were performed in serum-free DMEM supplemented with glutamine and antibiotics (DMEM++). For analysis of multicycle replication kinetics cells were seeded in 12-well plates and grown to 80 % confluency and either treated with protease inhibitors E64d (20 µM), BAPA (50 µM), MI-1148 (50 µM) 1 h prior to infection in DMEM supplemented with 3 % FCS, glutamine, and antibiotics (3 % DMEM+++) or remained untreated. Cells were then inoculated with virus at an MOI of 0.005 for Huh-7 and VeroE6 cells and MOI 0.0005 for Vero-TMPRSS2 cells in DMEM++ for 1 h, washed with PBS, and incubated in 3 % DMEM+++ with or without addition of protease inhibitors or DMSO to the medium for 72 h. At 16, 24, 48, and 72 h post infection (p.i.), supernatants were collected, and viral titers were determined by tissue culture infection dose 50 % (TCID_50_) titration as described below.

### 4.8 Virus titration (Tissue culture infection dose 50 (TCID_50_))

Viral supernatants were serial diluted in DMEM++. Each infection time point was titrated in four replicates from 10^1^ to 10^11^. Subsequently, 100 μl of each virus dilution were transferred to VeroE6 cells grown in 96-well plates containing 100 μl 3 % DMEM+++ and incubated for 72 h. Viral titers were determined with Spearman and Kärber algorithm described in reference (Hierholzer and Killington, 1996).

### 4.9 Reporter genome entry assay with transcription and replication-competent virus-like particles (trVLPs)

Transcription and replication-competent virus-like particles (trVLPs) containing a renilla luciferase encoding monocistronic reporter mini genome were generated in HEK293 cells as described in (Hoenen et al., 2006). The trVLPs were harvested from the cellular supernatant by ultracentrifugation at 40.000 rpm for 2 h on a 20 % sucrose cushion. The Huh-7 and VeroE6 cells were pre-transfected upon seeding with VP30, VP35, NP and L as well as a pGL4 firefly luciferase plasmid for normalization 24 h prior to trVLP infection. The cells were grown to a confluency of 80 % and either treated with protease inhibitors E64d (20 µM), BAPA (50 µM), MI-1148 (50 µM) 1 h prior to infection in 3 % DMEM+++ or remained untreated. Subsequently, cells were infected with trVLP preparation for 2 h in 600 µl DMEM++ and afterward supplemented with 3.5 ml 3 % DMEM+++ with or without the respective protease inhibitor for further 72 h incubation. Cell pellets were harvested, lysed and luciferase activity was measured with commercial kits (PJK).

### 4.10 Co-localization studies using confocal microscopy

For co-localization studies 4 × 10^4^ Huh-7 cells were seeded onto collagen-coated 12 mm cover slips in a 24-well plate. After 24 h cells were transfected with 600 ng of pCAGGs-TMPRSS2-FLAG in 10 % DMEM+++ and incubated at 37 °C and 5 % CO_2_ for 24 h. Subsequently, the cells were fixed either with 4 % PFA or with methanol/acetone (1:1), depending on the primary antibody, for 15 min at RT or on ice, respectively. The PFA-fixated cells were then incubated with 0.1 M glycine for 10 min and permeabilized with 0.1 % Triton-X-100 in PBS for 20 min. Cells were blocked for 1 to 2 h in immunofluorescence (IF)-blocking buffer (2 % BSA, 5 % glycerin, 0.2 % Tween 20 in PBS) and then incubated with primary antibodies against EEA1, Rab7, CD71 or TGN38 and a FLAG-specific antibody in IF-blocking buffer for 1.5 h at RT. Subsequently, the cells were incubated with Alexa-488- or Alexa-568-conjugated secondary antibodies for 45 min together with 4′,6-diamidin-2-phenylindol (DAPI) for a counter staining of cellular nuclei. The coverslips were mounted with fluoroshield onto glass slides and analyzed by confocal laser scanning microscopy (CLSM Leica TCS SP5 II, Leica Microsystems, Wetzlar (Germany)). Co-localization was determined by image analysis using FIJI software and the Coloc2 program.

## CRediT authorship contribution statement

**Dorothea Bestle:** Writing – review & editing, Writing – original draft, Validation, Methodology, Investigation, Data curation, Conceptualization. **Linda Bittel:** Investigation, Formal analysis, Data curation. **Anke-Dorothee Werner:** Methodology, Investigation, Data curation. **Lennart Kämper:** Resources, Methodology, Investigation. **Olga Dolnik:** Writing – review & editing, Resources, Methodology. **Verena Krähling:** Writing – review & editing, Resources, Methodology. **Torsten Steinmetzer:** Writing – review & editing, Resources, Funding acquisition. **Eva Böttcher-Friebertshäuser:** Writing – review & editing, Writing – original draft, Validation, Supervision, Resources, Methodology, Funding acquisition, Formal analysis, Conceptualization.

## Declaration of competing interest

The authors declare that they have no known competing financial interests or personal relationships that could have appeared to influence the work reported in this paper.

## Data Availability

Data will be made available on request.
